# Advanced Peptide Nanomedicines for Bladder Cancer Theranostics

**DOI:** 10.3389/fchem.2022.946865

**Published:** 2022-08-05

**Authors:** Sheng Zeng, Xiaodi Feng, Shaoqiang Xing, Zhaoliang Xu, Zhizhao Miao, Qian Liu

**Affiliations:** ^1^ Department of Urology, Tianjin First Central Hospital, Tianjin, China; ^2^ Department of Urology, Qingdao Hospital of Traditional Chinese Medicine (Qingdao Hiser Hospital), ShanDong, China; ^3^ Department of Urology, Weihai Central Hospital, ShanDong, China; ^4^ Department of Urology, First Central Clinical College, Tianjin Medical University, Tianjin, China; ^5^ School of Medicine, Nankai University, Tianjin, China

**Keywords:** peptide, bladder cancer, diagnosis, treatment, nanomedicines

## Abstract

Cancer is still a global public health problem. Although remarkable success has been achieved in cancer diagnosis and treatment, the high recurrence and mortality rates remain severely threatening to human lives and health. In recent years, peptide nanomedicines with precise selectivity and high biocompatibility have attracted intense attention in biomedical applications. In particular, there has been a significant increase in the exploration of peptides and their derivatives for malignant tumor therapy and diagnosis. Herein, we review the applications of peptides and their derivatives in the diagnosis and treatment of bladder cancer, providing new insights for the design and development of novel peptide nanomedicines for the treatment of bladder cancer in the future.

## Introduction

One recent report from the World Health Organization’s International Agency for Research on Cancer (IARC) released the latest global cancer burden data, showing that 4.57 million cancer cases and 3 million resultant deaths increased in 2020 in China. Among them, bladder cancer is one of the common urinary malignancies and ranks among the top ten cancers in terms of morbidity and mortality. Bladder cancer is one of the most expensive cancers to cure because of its high recurrence rate (Barani et al., 2021). Although new techniques involving radiotherapy, immunotherapy, chemotherapy, etc., are blossoming in the treatment of bladder cancer (Booth et al., 2018; Tree et al., 2018; Wołącewicz et al., 2020), their toxic side effects and high costs limit their broad applications in clinical applications. Early diagnoses, including the examination of circulating tumor cells, CT scan, magnetic resonance imaging, positron emission tomography, bone scan, chest X-ray, etc., are crucial for the diagnosis and treatment of bladder cancer (Todenhöfer et al., 2018; van der Pol et al., 2018; Wu et al., 2018), but their disadvantages, such as nonspecificity, heterogeneity, and excessive detection, still limit their potential clinical applications (Faba et al., 2019). Cystoscopic biopsy can improve the diagnostic accuracy, but it is difficult to identify superficial mucosal lesions such as carcinoma *in situ*, and it is invasive. Abscission cytology is a standard non-invasive test for the diagnosis and monitoring of bladder cancer. It has the disadvantage of being insensitive to low-grade tumors and depends on accurate diagnosis by the pathologist (DeGeorge et al., 2017).

The primary purpose of drug delivery is to send enough drug payloads to the lesion sites while minimizing their exposure to healthy tissues. To improve the specificity and pharmacokinetics of anticancer drugs and avoid the side effects of traditional therapies, two main strategies involving drug carriers and covalent modifications are widely used. Drug carriers such as nanoparticles and hydrogels can protect drugs from the external environment before on-demand release when they reach lesion sites. Meanwhile, the physical and chemical properties of the drug carriers significantly determine their biological distributions (Fan et al., 2021; Huang et al., 2021). Covalent modifications enable temporarily masking or limiting the bioactivity of the drugs and confer them with the desired pharmacokinetics (Lin et al., 2019; Cooper et al., 2021; Yang et al., 2021). It is noteworthy that both the abovementioned strategies can alleviate the burden of drug metabolism and improve the therapeutic effects of the original drugs. Among numerous drug molecules, peptides are highly competitive candidates for the treatment of bladder cancer because of their small sizes, high specificity, low systemic toxicity, etc. In addition, the diagnosis of bladder cancer mainly depends on pathology and imaging examinations, while the detection accuracy is still low. Using specific biomarkers on bladder tumor cells, peptide nanotechnology can significantly improve the sensitivity and specificity for the diagnosis of bladder cancer. (Pan et al., 2014; Tummers et al., 2017).

## Peptide-Instructed Tumor Diagnosis

Magnetic resonance imaging (MRI) is a noninvasive technique for tumor diagnosis in current clinical medicine. Although it has been shown that MRI has the ability to display three-dimensional anatomical details without injury and provide high spatial resolution without invasiveness, MRI is still less sensitive than fluorescence imaging for monitoring tiny tissue damage, cellular activity, molecular activity, etc (Chandra et al., 2010; Schroeder, 2008). Therefore, the development of new contrast agents is expected to enable the improvement of the detection accuracy of MRI. Paramagnetic Gd^3+^ complexes and superparamagnetic iron oxide (SPIO) nanoparticles are two widely used contrast agents in MRI detection. Compared with the paramagnetic Gd^3+^ complex, SPIO is a better alternative to MRI contrast agents, of which the signal contrast is several orders of magnitude higher than that of the traditional Glacki contrast agent (Jun et al., 2008). In a study of human bladder tumors, the researchers reported that 1.5T magnetic resonance imaging using SPIO as the contrast agent realized *in situ* detection of malignant tumors with a small size to ∼4 mm, while it was unable to effectively distinguish the depth of tumor invasion into the bladder walls (Beyersdorff et al., 2000). The main reason is that the cellular internalization levels of SPIO are limited, and less than 1% of SPIO is internalized by nonspecific endocytosis pathways (Moore et al., 2001). Due to the great promise of SPIO in MRI applications, researchers have endeavored to develop a variety of SPIO conjugates to enhance its cellular uptake ratio, in which cell-penetrating peptides (CPPs), such as R11, are considered to be one of the best transporters to improve the active internalization of nanoparticles into target cells (Hsieh et al., 2011; Ding et al., 2017). Ding et al. recently also developed an SPIO nanoparticle whose surfaces are unctionalized with bladder cancer-specific fluorescein isothiocyanate (FITC) labeled cell-penetrating peptide (CPP) -polyarginine peptide (R11) for active targeting and imaging for bladder cancer, respectively. Their study showed that SPIO-R11 nanoparticles can be internalized by T24 cells in a dose-dependent manner, and that SPIO-R11 internalized dose is higher than that of SPIO itself, since R11 is a cell-permeable peptide that enables efficient drug delivery. Transmission electron microscopy (TEM) results indicated that SPIO-R11 is mainly located in cellular vesicles and lysosomes, but no signals in the nucleus were found. Due to the cellular specificity of SPIO-R11, the uptake of nanoparticles into bladder cancer cells was significantly higher than that of immortalized bladder epithelial cells. In addition, SPIO-R11 had a lower T2 relaxation time in MRI than SPIO. These results suggested that SPIO-R11 has great potential as a targeted contrast agent for the diagnosis and treatment of bladder cancer (Ding et al., 2017)**.**

Tumor cells are mutated from normal cells, of which the signal transduction pathways are significantly different from those of normal cells. Therefore, many signaling regulators or regulatory proteins are overexpressed in tumor cells and can be used as specific targets for tumor diagnosis (Oh and Bang, 2020; Wilson et al., 2021). Recently, targeted peptides have attracted intense attention because they can specifically bind with receptors on tumor cells. By conjugating with radioactive or fluorescent probes, scientists have prepared a variety of peptide probes to specifically orient and image tumors (Ciobanasu, 2021; Kwak et al., 2021; Lu et al., 2021; Sonju et al., 2021; Wang et al., 2021). For instance, Wei et al. recently constructed a loaded nanoscale oxygen generator (PLZ4@SED) by conjugating superparamagnetic iron oxide nanoparticles (SPIOns) with peptide motifs specific to bladder cancer cells. PLZ4@SED showed good tumor targeting and permeability to patient-derived bladder cancer cells. Meanwhile, they illustrated that the presence of PLZ4@SED can improve the contrast of MRI and promote chemotherapeutic efficacy by producing oxygen through the Fenton reaction to relieve hypoxia. It was also reported that PLZ4@SED presented great potential in the diagnosis and treatment of bladder cancer (Lin et al., 2021). Sweeney et al. (2017) demonstrated one successful application of mesoporous silica nanoparticles (MSN), which are functionalized using a bladder cancer-specific peptide CyC6, as the magnetic resonance contrast agent. Due to the effective binding of the modified MSN to tumor cells, tumor boundaries were much clearer in the T1-and T2-weighted MRI and fluorescence cystoscopic inspections compared to the traditional technique.

Cystoscopy is one gold standard for the diagnosis of bladder cancer. However, cystoscopy is an invasive and costly technique, and it is difficult to detect flat malignancies using this technique. Meanwhile, urine cytology is low-sensitivity for detecting low-grade lesions, of which the detection accuracy is highly dependent on the experience of the cytopathologists (Grossman et al., 2006; Alfred Witjes et al., 2017; Babjuk et al., 2017). Recently, several potential biomarkers have been identified that could potentially provide noninvasive and objective approaches for the detection of bladder cancers (Kluth et al., 2015). Lee et al. presented one peptide conjugate consisting of fluorescein and the peptide sequence of CSNRDARRC. They reported that the peptide conjugate can specifically bind to bladder cancer tissue using frozen sections. Meanwhile, the peptide conjugate could selectively bind to bladder tumor epithelial cells when it was injected into the bladder cavity using a tumor-bearing rat model. Furthermore, Lee et al. found that the peptide conjugate had the ability to indicate bladder tumor cells in urine, presenting great potential to be exploited as a real-time diagnostic probe to detect bladder cancer (Lee et al., 2007). Prothrombin activators (TSPs) can prevent angiogenesis in a variety of pathological conditions. Some structural domains and peptide derivatives of TSP-2 enable the promotion of angiogenesis in BC tissues. 4N1K (KRFYVVMWKK), derived from the C-terminal cell-binding domain of TSP-2, plays an important role in the pathology and prognosis of bladder cancer. Using the hematoxylin-eosin (H&E) staining technique to examine tumor tissues from bladder cancer patients, Nakamura et al. verified that 4N1K was significantly correlated with the tumor apoptosis index and microvascular density but negatively related to T stage, metastasis and tumor grade; promising 4N1K may be a useful biomarker and a new therapeutic target for UC-UUT patients (Nakamura et al., 2019).

## Peptide-instructed Local Chemotherapy

**FIGURE 1:**
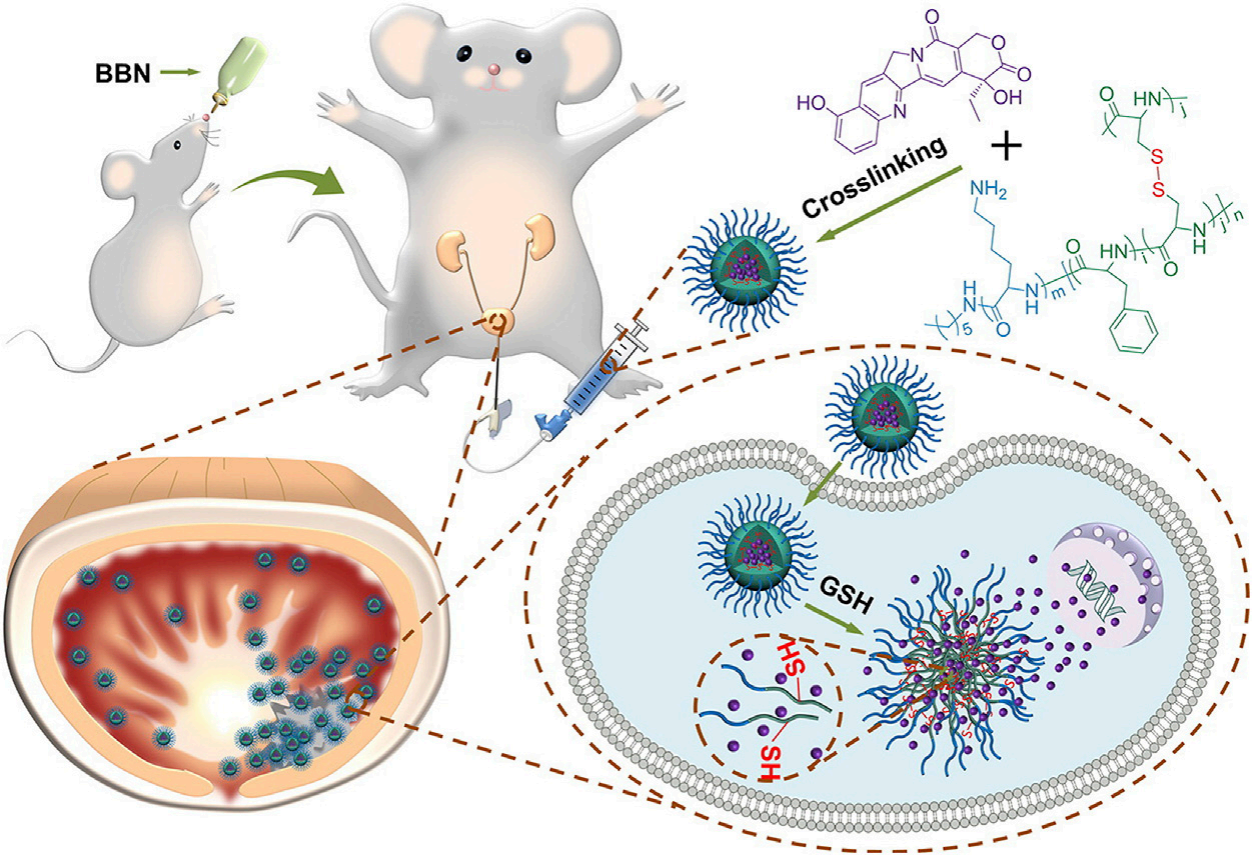
After intravesical instillation of NG/HCPT, the selective accumulation of nanodrugs in tumor tissue finally releases HCPT triggered by GSH(Guo et al., 2017).

Transurethral resection combined with chemotherapeutic infusion is the standard treatment protocol for nonmuscular invasive bladder cancer. However, the low bioavailability (GuhaSarkar and Banerjee, 2010) and short retention period of the current chemotherapeutic drugs (Tyagi et al., 2006; Wirth et al., 2009) restricted their exposure time at the lesion sites. Along with the advancement of nanotechnology, nanocarrier drug delivery systems show advantages in solving these problems. Guo et al. (2017) designed and synthesized a kind of positively charged intelligent peptide nanocarrier cross-linked with disulfide bonds [i.e., PLL-P (LP-*co*-LC). They prepared one nanogel system (NG/HCPT) using this nanocarrier by artificially loading 10-hydroxycamptothecin (10-HCPT). Compared with free 10-HCPT, NG/HCPT not only has a higher drug loading rate, longer retention time, and stronger tissue penetration ability but can also accurately and rapidly release 10-HCPT into bladder cancer cells, significantly enhancing the corresponding antitumor effects and reducing the side effects (Figure 1). In 2020, Guo et al. further synthesized a new R_9_-polyethylene glycol poly (L-phenylalanine-L-cysteine) nanogel (R_9_-PEG-P (LP-*co*-LC)] based on NG/HCP, which can improve the adhesion and permeability of chemotherapeutic drugs. They prepared the R_9_NG/HCPT nanogel using 10-HCPT as a model drug. The morphology of R_9_NG/HCPT is similar to that of an octopus with a spear. Highly positively charged R_9_ with strong membrane penetrability can help R_9_NG/HCPT pass across the bladder walls and enhance its cellular adhesion interactions through nonspecific and electrostatic interactions, thus enabling prolonged exposure to chemotherapeutic drugs at the lesion sites. This system significantly improved the tumor suppression efficiencies of 10-HCPT in both *in situ* mice and rat tumor models, suggesting great potential in the local chemotherapy of bladder cancer (Guo et al., 2020).

Polymeric micelles constructed using amphiphilic block copolymers have been widely explored in recent decades due to their high drug loading efficiency, long cycle time, well-controlled release ability, and good targeting properties (Xiao et al., 2012). Recently, Zhou et al. developed an amphiphilic diblock copolymer poly (ε-caprolactone)-b-polyoxyethylene (PCL-b-PEO) containing integrin targeting motif c (RGDfK) and imaging dye FITC. The copolymer assembled into micelles and strongly interacted with bladder cancer T24 cells. After encapsulation with doxorubicin (DOX), the micelle could efficiently prevent the proliferation of T24 cells and was expected to be used as a nanoscale drug delivery system for bladder perfusion chemotherapy (Zhou et al., 2013).

The combination usages of two or more drugs showed great advantages in cancer treatments involving improving therapeutic efficacy, lowering side effects, and preventing drug resistance, which are promising strategies for the treatment of refractory cancers. The positively charged adhesive chitosan-polymethacrylic acid (CM) nanocapsules loaded with DOX and cisplatin modified with peptide (Pt-Aly) presented high drug loading efficiency and sustained drug release properties. Meanwhile, CM nanocapsules can be firmly attached to the surface of the bladder cavity, prolong the retention time of the payload in the bladder, and have the effect of synergistically killing UMUC3 bladder cancer cells. In addition, CM nanocapsules have no obvious damage to the urothelium, which is expected to cooperate with intravesical chemotherapy in the treatment of non-muscle invasive bladder cancer (Lu et al., 2016). Overall, the intelligent peptide nanogel systems have much more powerful retention efficiency and permeability, providing a promising drug delivery platform for local chemotherapy of bladder cancer.

## Peptide-assisted Systemic Chemotherapy

**FIGURE 2:**
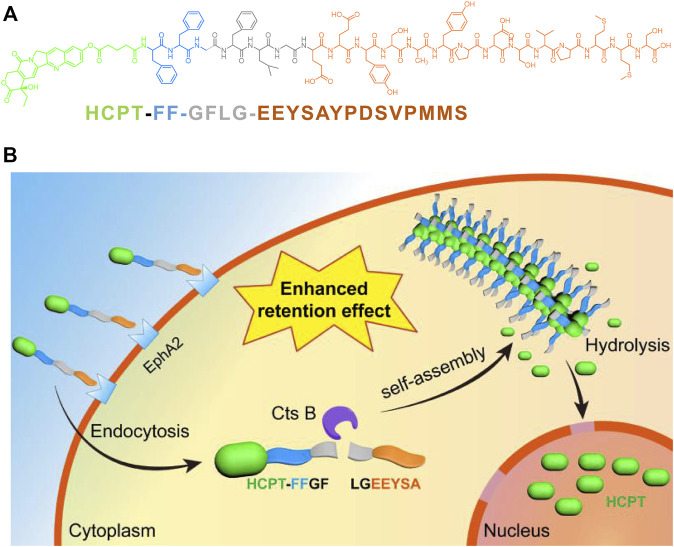
**(A)** The molecular structure of HCPT-FF-GFLG-EEYSAYPDSVPMMS; **(B)** The illustration of the bladder tumor cell targeting, intracellular fibrillation and drug release of HCPT-FF-GFLG-EEYSAYPDSVPMMS(Zeng et al., 2021a).

Systemic chemotherapy is one of the dominant techniques used to treat musculoskeletal invasive bladder cancer (Calabrò and Sternberg, 2009; Yin et al., 2016). However, nonspecific distributions of traditional chemotherapeutic drugs in human bodies have caused severe toxicity to normal tissues, including liver and kidney organs, bone marrow, gastrointestinal tract tissues, etc., and significantly limited their clinical applications. Therefore, researchers are endeavoring to develop new drug delivery systems that can transport the chemical drugs into the desired sites to improve their therapeutic effects (Cheng et al., 2019; Sonju et al., 2021). Peptide-drug conjugates are promising prodrugs for the treatment of cancer that combine one or more traditional chemical drugs with short peptides through biodegradable linkers. This prodrug strategy can uniquely and specifically employ the bioactivity and self-assembling properties of short peptides to enhance the therapeutic efficacy of traditional drugs (Cooper et al., 2021). Zeng et al. (2021b) recently developed one short-peptide prodrug, HCPT-FF-GFLG-EEYASYPDSVPMMS, consisting of 1) a self-assembling motif (i.e., -FF-); 2) an EphA2 targeting sequence on T24 cancer cells (i.e., YSAYPDSVPMMS); and 3) one short peptide linker responsive to the CtsB enzyme (i.e., GFLG). They found that this prodrug could be efficiently encapsulated by T24 cells and cleaved intracellularly by CtsB, resulting in nanofibrils in T24 cells (Figure 2). The formation of nanofibrils loaded with HCPT prolonged its circulation period *in vivo*. Moreover, this prodrug system could precisely deliver HCPT into T24 cancer cells, reduce its accumulation in normal tissue and lower the side effects. Pan et al. prepared one kind of nanomedicine, DC-PNM-PTX, in which one bladder targeting peptide sequence, PLZ4, one polymeric micelle, and the chemical drug paclitaxel (PTX) were involved. They reported that DC-PNM-PTX could specifically target bladder cancer cells, prevent bladder tumor growth in a xenograft tumor model, and efficiently prolong mouse survival compared to unmodified PTX. Nanomaterials modified with multiple ligands targeting cell membrane receptors play positive roles in tumor therapy, which is beneficial for reducing the toxicity and side effects of traditional chemotherapy and improving antitumor outcomes (Pan et al., 2016).

## Peptide-instructed Gene Therapy

Gene therapy is a revolutionary technique that directly uses therapeutic genes to treat various diseases. As one alternative to traditional treatments (Dunbar et al., 2018; High and Roncarolo, 2019), the first clinical trial of gene therapy was approved in 1989, and nearly 2,600 trials have been completed or are under their ways worldwide until now (Ginn et al., 2018). However, it is still challenging to direct the genes into targeted cells without damaging other cells. It has been shown that virus-like particles (VLPs) from human JC polyomavirus (JCPyV) can package and deliver exogenous DNA into sensitive cells for gene expression (Chang et al., 1997). To improve the specificity of gene therapy, Lai et al. (2021) conjugated SPB peptides targeting bladder cancer cells onto JC polyomavirus (JCPyV) virus-like particles (VLPs) and succeeded in the delivery of the suicide gene thymidine kinase. Both *in vitro* and *in vivo* experiments illustrated that the suicide gene was only expressed in human bladder cancer cells but not in lung cancer and neuroblastoma cells that were sensitive to JCPyV VLP infection, implying the great specificity of VLP-SPBs. Meanwhile, the gene transduction efficiency of VLP-SPBs is approximately 100-fold that of the VLP itself. The binding of JCPyV VLPs with specific peptides can improve their original affinities and change the expression directions of the packed genes. Moreover, VLP-SPBs presented the ability to selectively prevent the growth of bladder tumors but had no significant inhibitory effects against lateral lung tumors. In general, gene therapy is one flourishing technique to treat various diseases, and malignancies are their main enemy. The applications of the targeted peptide delivery systems enable artificial control and regulation of gene expression at the cellular level, thus succeeding in disease treatments but not affecting normal tissues and organs.

Epidemiological data have shown that more than 50% of human malignancies, including bladder cancer, are related to mutations in the p53 gene (Hainaut et al., 1997). Mutant p53 protein enables the acceleration of tumor formation and metastasis and is associated with resistance to radiotherapy and chemotherapy, as well as poor prognosis (Al-Sukhun and Hussain, 2003). The functional restoration of p53 protein can promote the expression of downstream genes to block cell cycles or induce cell apoptosis, resulting in the suppression of tumor progression. It has been shown that one C-terminal peptide sequence (p53c) can restore the binding ability to specific DNA sequences and the transactivation function of the mutant p53 gene, leading to p53-dependent apoptosis of tumor cells (Selivanova et al., 1997). However, due to the lipophilicity of biological membranes and their roles as biological barriers to defeat exterior enemies, many synthetic compounds cannot cross cell membranes. R11 can be specifically captured by bladder and prostate tissues and is promising for use as a drug or probe carrier for the treatment and detection of upper urinary tract tumors (Hsieh et al., 2011; Ding et al., 2017). Zhang et al. showed that the synthetic peptide R11-p53c can be effectively and preferentially delivered into bladder cancer cells, resulting in the reactivation of the p53 gene and inhibition of tumor growth. More interestingly, R11-p53c also presented excellent antitumor effects in primary and metastatic tumor models, which could prolong the survival period while having no significant systemic toxicities. In addition, their study also illustrated that R11-p53c could prevent the growth of both mutant and recombinant p53c tumor cells but had no significant inhibitory effects on normal cells. It was also noted that transcriptional levels of several p53 target genes were upregulated after treatment with R11-p53c. Overall, R11-p53c has the potential to treat both primary and metastatic bladder cancer and should be a promising therapeutic agent for the treatment of upper urinary tract tumors. (Hsieh et al., 2011).

## Peptide-mediated Photothermal Therapy

**FIGURE 3:**
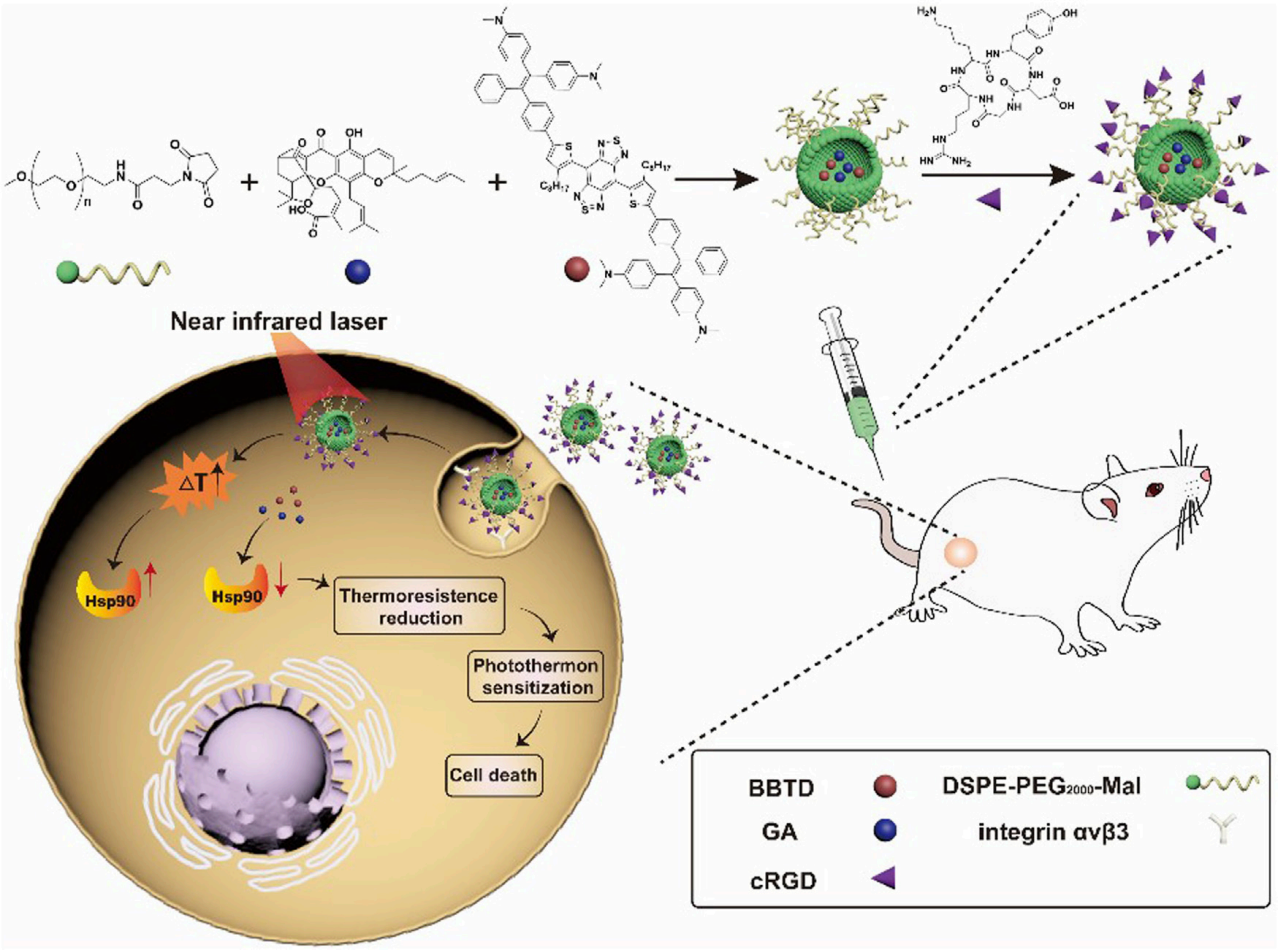
Schematic illustration of synthesizing BBTD + GA/PEG-cRGD nanoparticles for photohyperthermia therapy of MIBC(Zeng et al., 2021a).

Photothermal therapy (PTT) is a highly promising strategy to defeat malignancies that mainly utilizes photothermal materials to convert light energy into heat *in situ*, finally raising the local temperature to result in cell apoptosis and tumor killing (Gao et al., 2019; Chen et al., 2020; Jiang et al., 2020). By taking advantage of photothermal conversion, PTT has been widely used in a variety of tumor treatments, and some of them are under clinical trials (Timko et al., 2010; Chen et al., 2014). One crucial issue for PTT applications is to develop carrier materials with good selectivity to tumor cells. Tao et al. (Tao et al., 2019) loaded folate-modified vincristine into polydopamine-coated Fe_3_O_4_ (Fe_3_O_4_@PDA-VCR-FA SPs) and applied them for the treatment of bladder cancer. PDA shells can not only improve colloid stability and biocompatibility but also enhance photothermal effects and prolong the blood circulation period. The half-life period in blood and the tumor retention rate of Fe_3_O_4_@PDA-VCR-FA SPs are 2.83 h and 5.96% ID g^−1^, respectively, which are significantly improved compared with those before folic acid modification. The superparamagnetism of Fe_3_O_4_ and the loading of vincristine enable arming Fe_3_O_4_@PDA-VCR-FA SPs with nuclear magnetic resonance imaging (NMRI) and chemotherapy abilities. With the further help of near-infrared laser-triggered photothermal therapy, Fe_3_O_4_@PDA-VCR-FA SPs can completely remove bladder cancer and prevent its recurrence. Moreover, no obvious toxicity to the liver, kidney or other organs was detected through biochemical and pathological tests, suggesting the good biocompatibility of Fe_3_O_4_@PDA-VCR-FA SPs. Zeng et al. (2021b) recently reported a novel RGD-mediated photosensitive drug-peptide conjugate (BBTD + GA/PEG-RGD) for the treatment of musculoskeletal invasive bladder cancer. This system can specifically target integrin α_v_β_3_ outside the membrane of bladder cancer. Meanwhile, the system can prevent the overexpression of heat shock protein 90 and reduce the resistance of cancer cells to heat stress, finally succeeding in low-temperature PTT with great antitumor properties (Figure 3). Furthermore, the results of animal experiments showed that this system had advantages involving 1) good tumor targeting ability and stability; 2) less thermal damage to normal tissue; 3) great therapeutic effects against musculoskeletal invasive bladder cancer; and 4) a longer survival period compared to the control groups. Low-temperature PTT is highly effective in preventing tumor growth without damaging normal tissues, promising great clinical applications for optical tumor therapy in the future.

## Therapeutic Peptides

**FIGURE 4:**
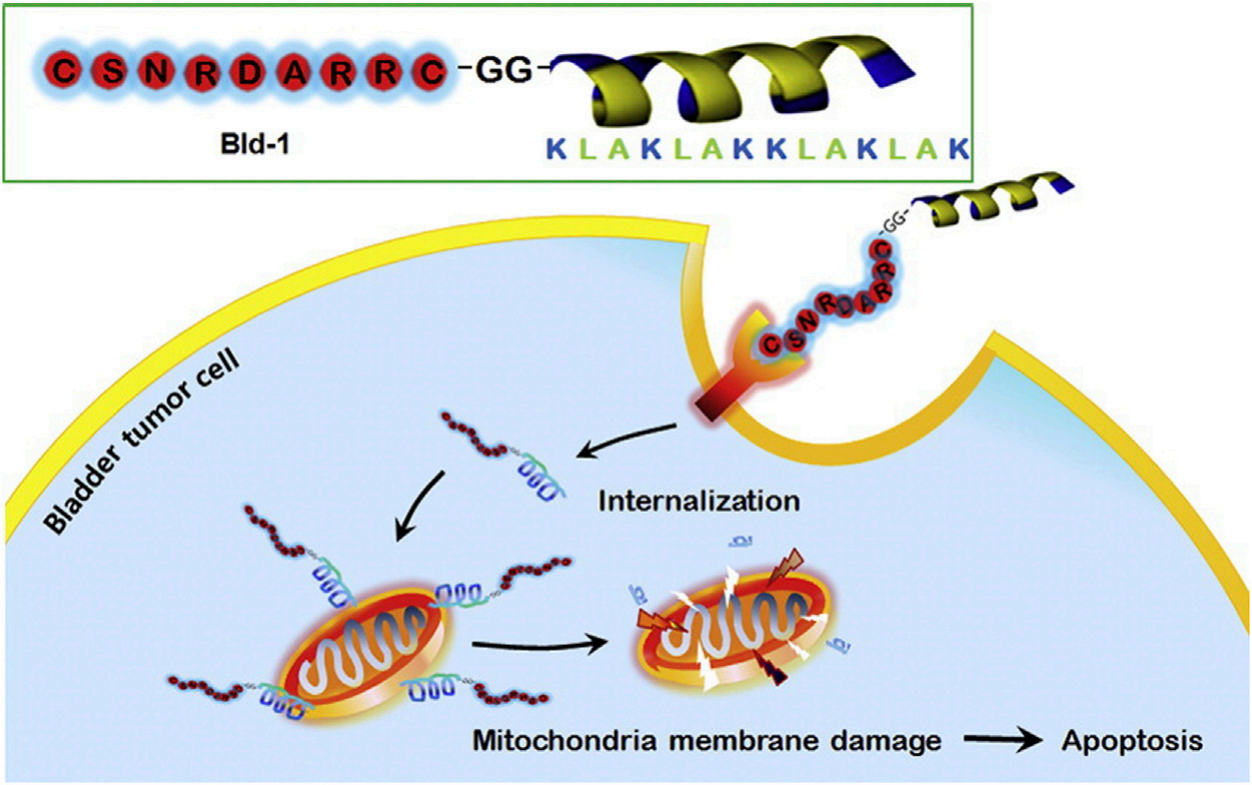
Bld-1-KLA destroys the mitochondrial membrane and induces apoptosis.

Mitochondria play an important role in apoptotic death (Bock and Tait, 2020), and some anticancer agents can destroy mitochondrial functions and induce tumor cell apoptosis (Vasan et al., 2020). One typical example is the cationic amphiphilic peptide KLAKLAKKLAKLAK (i.e., KLA). KLA is a natural antibacterial peptide that can bind and damage negatively charged bacterial membranes. Normally, KLA does not damage eukaryotic membranes and has no toxicity to eukaryotic cells. However, internalized KLA can rupture the mitochondrial membrane, resulting in cytochrome C release and cell apoptosis (Huang et al., 2017). KLA is always conjugated with transmembrane peptides (CPPs) to promote its internalization efficiency by tumor cells; however, the conjugated KLA-CPPs also have high cytotoxicity to normal cells because of their nonspecific interactions (Wang et al., 2016). To overcome the potential nonspecific interactions, Jung et al. designed and synthesized a mixed peptide (Bld-1-KLA) consisting of 1) a targeting peptide to bladder cancer cells CSNRDARRC (Bld-1) and 2) an effector peptide D-KLAKLAKKLAKLAK (KLA) that can destroy the mitochondrial membrane and induce apoptosis. Bld-1-KLA can selectively bind and internalize into bladder cancer cells to induce cell apoptosis without significant toxicity to other tumor cells and normal cells. After intravenous administration of Bld-1-KLA in the HT1376 tumor-bearing mouse model, it was shown that Bld-1-KLA had a higher tumor homing and inhibition ability than the control groups (Figure 4). Together, these results suggest that Bld-1-KLA is a promising targeted therapeutic against bladder cancer (Jung et al., 2016).

Fibroblast cytokine 9 (FGF9) is overexpressed in many cancer cells (Ren et al., 2016; Mizukami et al., 2017), and its targeted receptor FGFR3c is an important driver of bladder cancer progression (Iyer and Milowsky, 2013; Wang et al., 2020). The important role of FGFR3c makes it an important therapeutic target for the treatment of bladder cancer. Wang et al. (2020) reported one FGF9 binding peptide, P4, using the phage display technique. Meanwhile, they found that P4 is highly homologous to the immunoglobulin-like domain II-III (D2-D3) of FGFR3c using sequence comparison. Functional analysis showed that P4 had the ability to prevent the FGF9-induced aggressive phenotypes, including cell proliferation, migration, and invasion, and inhibit tumor progression by downregulating the MAPK and Akt cascade pathways. More importantly, FGF9 was found to be a potential driver of drug resistance in gastric and bladder cancer cells, in which the presence of P4 can increase the sensitivity of chemical drugs. In conclusion, Wang’s study identified a novel FGF9-binding peptide that may serve as a potential agent to treat malignancies with abnormally upregulated FGF9.

## Conclusion

In recent decades, significant success has been achieved in the diagnosis, treatment, and prevention of bladder cancer. However, bladder cancer is characterized by polycentricity, multiple occurrences, and recurrence, suggesting great challenges for its clinical treatment. With the development of modern biosynthesis technology, peptide nano drugs have become one of the hot spots in drug research. Compared with monoclonal antibody drugs, recombinant protein drugs and small molecule drugs, peptide nano drugs have the characteristics of simple spatial structure, significant curative effect and high safety, and have been widely used in the diagnosis and treatment of tumors. With the continuous progress of related technologies, the clinical application of peptide nano drugs is more and more in-depth, and the development space is broad. Peptides are promising for intracellular delivery of chemical drugs, DNA, siRNA, fluorescent molecules and nanoparticles. Compared with other chemical entities, peptides have the advantages of low molecular weight, low cost and good stability. At the same time, polypeptides can be easily modified to attach and enter tumor cells, and finally transport the goods to the desired and desired places. In general, peptide nanodrugs can improve tumor targeting and permeability, reduce systemic toxicity, reduce and prevent recurrence, shorten treatment time and reduce treatment cost. They are of great value for the clinical application of bladder cancer. Peptide drugs have outstanding advantages. With the continuous progress of biotechnology and peptide synthesis technology, peptide drugs have broad market development space and are expected to become one of the main drugs for cancer diagnosis and treatment (Lin et al., 2021).
